# Evaluating the Impact of a Medical School Student-Run Research Organization on Scholarly Activity

**DOI:** 10.7759/cureus.43067

**Published:** 2023-08-07

**Authors:** Jason T Bard, Hemasree Yeluru, Matvey V Karpov, David Mu

**Affiliations:** 1 Research, Eastern Virginia Medical School, Norfolk, USA

**Keywords:** step 1 pass fail, student research club, step 1 change, medical education, medical student research

## Abstract

Introduction

The United States Medical Licensing Examination Step 1 change to Pass/Fail scoring has motivated medical students to pursue more research opportunities. To support students, a student-led organization was created at an allopathic medical school, offering initiatives such as workshops, mentorship, and research projects. Here, we evaluate its impact on medical student research.

Methods

An observational survey study was conducted to assess students' research involvement and productivity and their sense of support, confidence, and comfort in pursuing research at an institution during the first two years of medical school. These variables were compared between three contiguous classes of students and between club members and non-members. Analyses included t-tests, Chi-square tests, and ANOVA, among others.

Results

Findings revealed that organization membership was associated with an increased number of research projects. Club members (*M*= 4.49) reported a significantly greater number of projects compared to non-members (*M*= 4.49) (*p*= 0.002). Students who had access to the organization during their preclinical years (*M*= 4.38) reported significantly more projects compared to students whose preclinical years were before the organization’s conception (*M*= 2.21) (*p*= 0.041). However, research productivity and feelings of support and confidence in research did not differ by class or club membership.

Conclusions

Club members engaged in a greater number of research projects as compared to non-members and students who had access to the organization during their preclinical years. The implementation of similar organizations at every medical school can allow more students to engage in scholarly work.

## Introduction

The United States Medical Licensing Examination (USMLE) Step 1 was introduced to American medical schools in 1992 and, for nearly three decades, had employed a numerical scoring system to evaluate students [1]. Beginning in January 2022, Step 1 was slated to utilize a pass or fail (P/F) scoring system, dramatically changing the playing field of medical education. Although Step 1 was originally unintended to be used as a screening tool for residency programs to filter applicants, in its latter decade of life, the exam ended up serving that exact purpose [2]. According to the National Resident Matching Program’s (NRMP) 2020 Program Director Survey, 90% of programs across all specialties rated the importance of an applicant’s Step 1 score as a 4 out of 5 when selecting which applicants to interview, highlighting the weight that program directors have placed on the exam since its conception [3]. By nature of this scoring change, students in the class of 2024 matriculated into medical school with a cloud of uncertainty looming overhead: how would this affect their education and, ultimately, their success in the residency matching process? 

While the impetus to use a P/F scoring system was primarily to alleviate stress and burnout among medical students, its aftermath still remains ambiguous for the next handful of graduating classes. One hypothesis is that a P/F system will improve the experience of medical students by decreasing the burden of the exam and subsequently improving their wellbeing [4]. An alternative perspective to consider is that without a numeric Step 1 score, medical students with a desire to stand out on their residency applications will feel pressured to pursue more community service, research, or leadership roles, therefore merely displacing the previous stress associated with Step 1. The perceived competitiveness of a medical student’s residency of choice has been shown to positively impact research productivity [5]. Among the several potential changes medical students might undertake, increasing their participation in scholarly activity and research is of particular interest. 

To support incoming preclinical (defined at this institution as the first and second years of medical school) medical students during this unclear transition period, we founded a student-led organization at an LCME-accredited U.S. allopathic medical school (Eastern Virginia Medical School in Norfolk, VA). This organization was termed the Eastern Virginia Medical School Research Society (ERS). We aimed to facilitate student participation in research by providing workshops, informational sessions, panel discussions, and journal clubs. Additionally, a mentorship program was established to offer students with minimal research experience personalized guidance in navigating research at the institution. As leaders of the club, we paired faculty with students who were interested in conducting research within their field. Overall, these initiatives provided an avenue for students to openly collaborate with their peers and form vital connections with the faculty, thereby advancing their involvement and interaction with research. Since its conception, our organization has been greeted with immense interest, with approximately 100 student members joining the organization annually (two-thirds of the average M.D. class size at this school).

One and a half years following the initiation of the club, we designed a study to evaluate the impact the organization had on medical students, specifically their research involvement, productivity, and confidence, during their first and second years of medical school. We also aimed to identify potential areas of improvement to enhance the efficacy and usefulness of the organization. 

The following are the specific questions our team sought to answer within our three overarching research questions:

1. Research involvement 

 a. Are more medical students participating in research projects (both within and outside the institution) during their first and second years?

 b. Are medical students participating in a greater number of research projects (both within and outside the institution) during their first and second years?

2. Research productivity

 a. Are more medical students producing scholarly work during their first and second years? Scholarly work includes abstracts, presentations, and publications.

 b. Are medical students producing a greater amount of scholarly work during their first and second years? Scholarly work includes abstracts, presentations, and publications.

3. Sense of support (SS) and confidence and comfort (CC)

 a. Do medical students report an increased level of support in conducting research at their institution?

 b. Do medical students report an increased level of confidence in conducting research at their institution?

## Materials and methods

Participants

**Figure 1:**
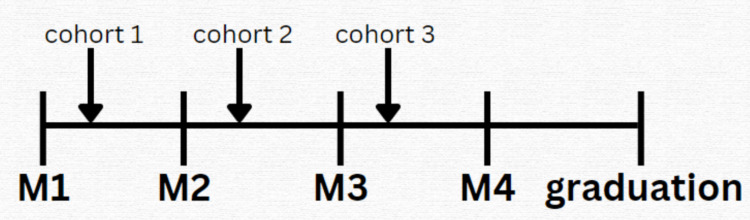
Point of each cohort at the time of club formation (10/2020) M1: first-year medical student; M2: second-year medical student; M3: third-year medical student; M4: fourth-year medical student

**Figure 2:**
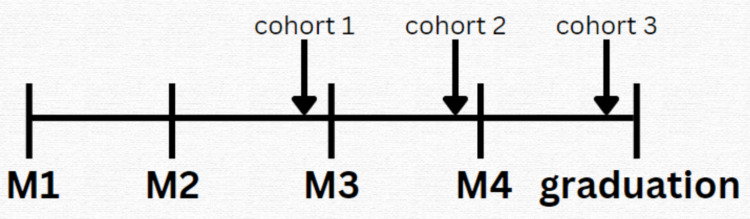
Point of each cohort at time of survey (04/2022) M1: first-year medical student; M2: second-year medical student; M3: third-year medical student; M4: fourth-year medical student

All students from the Doctor of Medicine (M.D.) classes of 2022, 2023, and 2024 of this institution were permitted to participate in this study, regardless of their involvement with the research organization or research in general. Participation in research is not a requirement of the M.D. program. Students from any other M.D. class (e.g., 2025) or a different program, such as Master of Public Health and Physician Assistant, were excluded from the study. The M.D. class of 2025 was excluded due to not having had sufficient time to complete and thus report research activity. In this paper, we refer to each graduating class as a cohort. We assigned the class of 2024 as cohort 1, the class of 2023 as cohort 2, and the class of 2022 as cohort 3. Cohorts 2 and 3 were included in this study to serve as comparison groups to cohort 1, as the club was not yet established until cohort 1 began medical school in 2020, which was after the majority of cohort 2 and 3’s preclinical years defined as the first two years of medical school. Figures 1, 2 depict the educational stage of each cohort during the time of club formation and the time of the survey, respectively.

Recruitment

To recruit participants, an email with a short description of the study and a link to an online survey was sent out to students within the aforementioned cohorts by a student affairs representative at the institution. A reminder email was sent out two weeks after the initial recruitment email. 

Survey administration

We created a self-reported questionnaire with 27 questions containing the following categories: demographics (e.g., class year, club member status, etc.), research involvement and productivity, sense of support, confidence, and comfort, and an optional free response section for qualitative comments and feedback from members of the organization (Refer to Appendix A for the full questionnaire). The survey was created and administered through REDCap, a secure online research application [6].

Before beginning the survey, respondents were instructed to refer to their curriculum vitae (CV) to minimize any recall bias. The first research question aimed to address “research involvement” to discern whether those students who responded to the survey participated in research at all during their preclinical years, in addition to the average number of projects they participated in. The second research question addressing “research productivity” assessed whether students produced any scholarly work such as abstracts, presentations, or publications during their preclinical years. The third research question aimed to measure subjective outcomes such as research support, comfort, and confidence. This was divided into “sense of support” and “comfort and confidence.” Both categories contained six questions, three of which were adapted from the Dental Student Research Inventory [7]. The survey responses were measured on a five-point Likert scale ranging from strongly disagree (1 point) to strongly agree (5 points). All data and email addresses were kept confidential by the study team and password-protected within REDCap. 

Response to the survey was incentivized with a $10 gift card raffle. Individuals who completed the survey were given the opportunity to opt into the raffle by providing their email address for contact purposes. One medical student on the research team was non-blinded to the participants' identities after the survey was closed for data collection to coordinate the distribution of gift cards. Twenty respondents were selected using a random number generator, and winners were contacted via email and provided with an electronic gift card. 

Data analysis

Due to the distribution of some of the data, both parametric and non-parametric analyses were conducted, including descriptive statistics, student’s t-test, Chi-square, Fisher’s exact, Kruskal-Wallis, Mann-Whitney U, and ANOVA. Data were analyzed using SAS 9.4 software (SAS Institute Inc., Cary, NC, USA). This study was approved by our institution's IRB (#21-11-NH-0259). 

## Results

Demographics and research involvement

A total of 116 medical students completed the survey. There were 63 entries from cohort 1 (54.3%), 32 from cohort 2 (27.6%), and 21 from cohort 3 (18.1%). Additionally, 45.7% of respondents (n= 53) were club members, of whom the majority were from cohort 1 (92.5%; n= 49). Less than half (43.97%) of respondents reported utilizing the organization’s resources. Only 9.48% of respondents reported having participated in a research project shared by the organization (n= 11). Most respondents reported having worked on at least one research project during their M1 or M2 years, with only 12.07% having not (n= 14). Most also reported having no publications as a first author (86.33%; n= 85). Additional details are highlighted in Table 1.

**Table 1:** General parameters of respondents M1: first-year medical student; M2: second-year medical student

Categorical Variables	n (%)
Class	
Cohort 1 (MD 2024)	63 (54.31)
Cohort 2 (MD 2023)	32 (27.59)
Cohort 3 (MD 2022)	21 (18.10)
Club membership	
Yes	53 (45.69)
No	63 (54.31)
Utilized club resources	
Yes	51 (43.97)
No	65 (56.03)
Participated in project via club	
Yes	11 (9.48)
No	105 (90.52)
Worked on at least one research activity in M1 or M2 years	
Yes	102 (87.93)
No	14 (12.07)
Number of publications as first author	
0	85 (86.33)
1	12 (11.76)
2	4 (3.92)
4	1 (0.98)

Research productivity

Respondents reported an average of 1.99 accepted abstracts (SD= 2.27), 1.46 posters and presentations (SD= 1.63), and 0.60 publications (SD= 1.16). Respondents reported an average of 2.09 years of research before medical school (SD= 1.69) and having worked on an average of 3.49 research projects during M1 or M2 years (SD= 3.04). The students’ mean sense of support (SS) was calculated as 3.35 out of 5 (SD= 0.80), and the mean comfort and confidence (CC) score was 3.45 (SD= 0.86). Shapiro-Wilk tests for normality were performed, indicating non-normal distributions for all continuous variables listed in Table 2.

**Table 2:** Research productivity, sense of support, and confidence and comfort IQR: interquartile range; SD: standard deviation

Continuous Variables	Min	Max	Median (IQR)	Mean (±SD)	Shapiro-Wilk test of normality W (p-value)
Years of research prior medical school	0	8	2.00 (2.00)	2.09 (±1.69)	0.88 (p<0.0001)
Number of projects during M1-M2 years	1	15	2.00 (3.00)	3.49 (±3.04)	0.77 (p<0.0001)
Number of abstracts accepted	0	11	1.00 (2.00)	1.99 (±2.27)	0.77 (p<0.0001)
Number of presentations	0	9	1.00 (1.00)	1.46 (±1.63)	0.72 (p<0.0001​​​​​​​)
Number of publications	0	7	0 (1.00)	0.60 (±1.16)	0.58 (p<0.0001​​​​​​​)
Number of publications as first author	0	4	0 (0)	0.24 (±0.62)	0.44 (p<0.0001​​​​​​​)
Likert Scale Variables	Min	Max	Median (IQR)	Mean ± SD	Shapiro-Wilk test of normality W (p-value)
Mean Sense of Support (SS)	1	5	3.33 (1)	3.35 (±0.80)	0.98 (p = 0.26)
Mean Comfort & Confidence (CC)	1	5	3.5 (1)	3.45 (±0.86)	0.96 (p = 0.003)

Research outcomes

Through Kruskal-Wallis (ANOVA) and Mann-Whitney U testing, it was discovered that among students who were involved in research, their graduating class and club membership were associated with an increased number of research projects and accepted abstracts. Specifically, club members reported a greater number of projects compared to non-members (p= 0.002; members M= 4.49, SD= 3.60; non-members M= 2.57, SD= 2.04). Among cohorts 1, 2 and 3, there was a significant difference in the number of research projects participated in during M1-M2 years (p= 0.019; cohort 1 M= 4.38, SD= 1.16; cohort 2 M= 2.40, SD= 1.26; cohort 3 M= 2.21, SD= 1.58). A post-hoc test determined that students from cohort 1 reported significantly more projects compared to cohort 3 (p= 0.041). However, there was no difference between club members and non-members in terms of the number of conference presentations, total publications, or first-author publications. Details can be found in Table 3.

**Table 3:** Comparison of club members vs. non-members SD: standard deviation

Continuous Variable	Club member (n=49)			Non-member (n=53)			
	Mean	Median	SD	Mean	Median	SD	p-value
Number of projects during M1 and M2 years	4.49	3.00	3.6	2.57	2.00	2.04	0.002
Number of abstracts accepted	2.47	1.00	2.73	1.55	1.00	1.65	0.07
Number of conference presentations	1.76	1.00	2.04	1.19	1.00	1.08	0.15
Number of publications	0.73	0.00	1.29	0.43	0.00	0.80	0.22
Number of publications as first author	0.31	0.00	0.71	0.15	0.00	0.36	0.26

Sense of support (SS) and confidence and comfort (CC)

The sense of support, confidence, and comfort scales were found to be reliable (α= 0.84 and α= 0.86, respectively), and the mean levels of each construct did not differ by graduation year or club membership status. 

Qualitative comments

Finally, common themes among qualitative comments included appreciation for the various initiatives that the organization provided for students, specifically for the frequent recruitment for available research projects. Some comments described barriers to research, including not being aware of the club. “I did not know [the club] was a resource I could be part of. The research lab I did take part in was detrimental to my time and mental health,” noted one respondent, highlighting the toll research can take on students already short on time to keep up with coursework. Another noted, “I think the toughest part is knowing where to start for research, and [the club] wasn't available when I was an M1.” Constructive feedback included finding “ways to expand and maximize [the club’s] research projects (i.e., how to find conferences on your own).” It was also indicated that the institution and organization should find ways to facilitate research publications to a greater degree in the future, especially due to the perception that more scholarly work is produced at other institutions. On the other hand, many positive comments were given as well, with a respondent writing, “[the club] did an amazing job connecting students with mentors and holding panels to generate more awareness about the research studies conducted at [the institution].” Another wrote, “I used [the club] to join my current lab, which has been a fantastic experience. I am very grateful that they offered to match students with labs.”

## Discussion

Our study explored the productivity of medical students conducting research before and after the foundation of a focused research organization. An attempt was made to bridge the gap between students and the institution by reducing barriers to obtaining research opportunities through mentorship, workshops, didactics, and networking. Study findings revealed that students involved in research, particularly those who are members of the club, have engaged in more research projects since the organization’s conception. In one study at a southern medical school, orthopedic research faculty were linked with medical students through a research committee. It was found that the number of first- and second-year students participating in research increased by 460% (5 to 28), along with a 780% increase (5 to 44) in research projects with medical student participation, indicating a clear benefit of having a research organization [8]. At another institution, the creation of an internal medicine interest group research program resulted in a significant increase in student research presentations, awards, and publications [9].

Qualitatively, participants in our study commented that the organization laid the foundation for students to feel supported and garner a sense of community, which could also explain the increased engagement in research projects. However, the amount of scholarly work, such as published abstracts or papers, was not significantly associated with either cohort or membership status. This could suggest that, despite an increase in students’ involvement with research, their work is not always being materialized or disseminated. Publications indicate a larger achievement, as compared to abstracts or presentations, on one’s curriculum vitae and are, therefore, an important factor to track. One possible explanation is that the survey data was collected based on students’ achievements during only the first half of medical school, which may have been too early for many to have completed their research projects and had their publications accepted. Perhaps if the survey had been administered at the time of graduation for all classes, a significant difference in research productivity could have been appreciated. Another consideration is the potential need for more research support at this institution. As some qualitative comments on the survey noted, the institution may still have room to expand in terms of research opportunities and ease of publishing. One option could be to establish a website that lists every research opportunity available at the medical institution, along with online discussion forums. This is an idea shown to be successful at a different institution’s student-led initiative for undergraduate medical research [10]. 

Another finding was that feeling supported and confident in conducting research was not higher among club members compared to non-members, which perhaps could be mitigated by offering more opportunities for mentorship and networking. A survey of osteopathic medical schools discovered that while nearly all colleges of osteopathic medicine offered medical student research electives, research funding, and symposia, only 62.5% offered structured research programs [11]. Continuing a structured, evidence-based research organization at our institution is likely to result in a tangible increase in perceived support in the coming years.

A limitation to recognize is how students could have pursued more research opportunities solely due to the change in Step 1 rather than due to the organization itself. Survey data was analyzed between club members and non-members to address this uncertainty; however, 92.5% of members were also members of cohort 1 (MD’ 24), which made it difficult to delineate the impact of membership, rather than class year, on scholarly activity. Comparing members and non-members only within cohort 1 was not performed and is a potential area of study for future years. There is also the possibility of selection bias, as students with a pre-existing interest in research are more likely to join a research-focused club and participate in research. As the club grows, a greater understanding of the evolving role that research plays in residency applications may further clarify our findings. 

Scholarship is fundamental to the advancement of medicine; over the past 50 years, several medical schools have begun to incorporate formal research programs and competencies into their curricula [12-13]. By emphasizing the importance of scholarly activity, these institutions have observed a rise in the number of students becoming academic physicians who practice evidence-based medicine [14]. While many distinguished institutions are moving towards adopting formalized research programs, most medical schools only offer informal avenues for students to conduct research. There is scarce literature highlighting the prevalence of student-run research organizations at medical schools; these organizations likely do exist but have not been analyzed for their efficacy. It is worthwhile to evaluate the presence of student-run research organizations in medical schools and understand their impact on student research, residency, and involvement in academia. Additionally, this study may influence other medical students to implement a similar organization at their respective institutions to promote research and scholarly activity. 

## Conclusions

The study findings revealed that students who were able to access research resources from the organization were more likely to participate in a greater amount of research. Other outcomes, such as the number of presentations and publications, in addition to a sense of confidence and support in research, were not found to be different among class cohorts or club membership. With the continued growth of the club through new iterations of leadership and a further understanding of the evolving role research will play in residency applications, we hope to see our institution and other institutions across the country expand opportunities for students to engage in and produce scholarly works with a sense of confidence and community.

## Appendices

Appendix A 

Survey

Pre-survey information: This survey is intended to gauge research participation and productivity among medical students at Eastern Virginia Medical School (EVMS) and has an estimated completion time of 10 minutes. Prior research experience is NOT required to take this survey; the only requirement is being an EVMS medical student in the MD class of 2022, 2023, or 2024. In order to ensure the most accurate data, please refer to your Curriculum Vitae (CV) or resume while reporting your number of research experiences in this survey. If desired, you may enter into a raffle for one of twenty $10 Starbucks gift cards upon completion of the survey. Thank you for your participation.

Demographics

1) Which EVMS MD class are you a part of?

-MD 2022

-MD 2023

-MD 2024

-Other (end survey)

2) Were you a previous Medical Masters student at EVMS?

-Yes

-No

3) How many years of research experience (includes time spent working on at least one research project in any discipline) did you have prior to starting medical school?

-*Type in Number*

4) Were you a member or participant of the EVMS Research Society (ERS) at any point during your M1 or M2 years?

-Yes

-No

5) Have you attended or utilized any of the information that the ERS provided from 2021 to 2022? (e.g., presentations, workshops, journal clubs, panels, mentorship, etc.) -Yes

-No

6) Have you directly been a part of any research project that was distributed through the ERS from 2021 to 2022? 

-Yes

-No

Research Involvement

7) Did you work on at least one research activity at any time during your M1 or M2 years?

-Yes

-No

8) What is the total number of research projects you participated in during your M1 and M2 years? (Only appears if respondent selects Yes to question 7)

-*Type in number*

Research Productivity

(Numeric answers; questions 9-12 only appear if respondent selects Yes to question 7)

During your M1 and M2 years…

9) How many of your research abstracts, if any, were accepted to a conference or meeting?

10) How many research projects did you present at a conference or meeting?

11) How many research projects were published in a peer-reviewed journal in which you were an author?

12) How many research projects were published in a peer-reviewed journal in which you were listed as first author?

13) Did you have an interest or make an effort to find research opportunities during your M1 or M2 years? (Only appears if respondent selects No to question 7)

Sense of Support & Confidence, and Comfort

(Five option Likert scale: strongly disagree to strongly agree (0 to 5))

(The following questions only appear if respondent selects Yes to question 7 OR selects Yes to question 13)

14) There are enough resources at EVMS to assist me in research. (SS)

15) Adequate facilities are available to me to conduct analysis and analyze data for research. (SS)*

16) I have easy access to literature (e.g., books, journals) to support my research. (SS)*

17) I received adequate guidance, help, or support whenever I faced any difficulties during research from staff members, my seniors, and/or my colleagues. (SS)*

18) There are people at EVMS who can help me find research opportunities. (SS)

19) I feel a strong sense of support surrounding my research endeavors. (SS)

20) I am confident in my ability to conduct research. (CC)

21) I feel comfortable conducting research. (CC)

22) I know where to go to find research opportunities. (CC)

23) After working on a research project during my time at EVMS, I felt it was a good experience. (CC)*

24) I have the necessary skill and knowledge to be able to write a scientific article. (CC)*

25) I was able to write up my research to my complete satisfaction by myself or after consulting my seniors. (CC)*

**Adapted from the Dental Students Research Inventory (DSRI) *[7].

Free Response

26) If you were involved in the EVMS Research Society to any extent, please let us know of any comments or advice you have for the club. (optional)

-*Type in*

Incentive

27) Thank you for completing the survey. If you would like to be entered into a raffle for one of twenty $10 Starbucks gift cards, please enter your email address below. (optional)

-Type in
